# Association of Periodontal Disease with Sleep Quality and Oral Health-Related Quality of Life: A Cross-Sectional Study

**DOI:** 10.3390/healthcare14162456

**Published:** 2026-08-09

**Authors:** Vedat Yüksekkaya, Ebru Sarıbaş, Ramazan Ağırağaç, Bülent Ulaştan, Hatice Ortaç

**Affiliations:** 1 Department of Periodontology, Faculty of Dentistry, Dicle University, Diyarbakır 21000, Turkey; ebruecesaribas@gmail.com (E.S.); rmznagiragac@gmail.com (R.A.); bulentulstn@gmail.com (B.U.); 2 Department of Biostatistics, Faculty of Medicine, Dicle University, Diyarbakır 21000, Turkey; haticeortac21@gmail.com

**Keywords:** periodontal disease, PSQI, OHIP-14, sleep quality, oral health-related quality of life

## Abstract

**Background/Objectives:** This study aimed to comprehensively evaluate the interaction between periodontal disease, sleep quality, and quality of life and to investigate the effects of the presence and severity of periodontal disease on individuals’ sleep patterns and oral health-related quality of life. **Materials and Methods:** A total of 195 individuals were included in the study. Participants’ sociodemographic data and dental habits were recorded. Sleep quality was assessed using the Pittsburgh Sleep Quality Index (PSQI), while the impact of oral health on quality of life was evaluated using the Oral Health Impact Profile-14 (OHIP-14). **Results:** A significant difference was observed in OHIP-14 scores among the groups (*p* < 0.001), with median values of 6, 13, and 22 in the healthy, gingivitis, and periodontitis groups, respectively. OHIP-14 scores also increased significantly with the stage and grade of periodontitis (*p* < 0.001). PSQI scores differed significantly among the groups (*p* < 0.001), with median values of 4, 8, and 10 in the healthy, gingivitis, and periodontitis groups, respectively. However, no significant differences were observed in total PSQI scores across the stages or grades of periodontitis. In addition, a strong positive correlation was found between total PSQI and OHIP-14 scores (r = 0.63, *p* < 0.001). Multiple linear regression analysis confirmed that periodontal status remained independently associated with both PSQI and OHIP-14 scores after adjusting for age, sex, education level, and smoking status, with periodontitis showing the strongest association in both models (PSQI: B = 5.03; OHIP-14: B = 15.80, both *p* < 0.001). **Conclusions:** A significant relationship was identified between periodontal disease, sleep quality, and oral health-related quality of life. Furthermore, the strong positive correlation between sleep quality and oral health-related quality of life suggests that deterioration in sleep quality may be associated with poorer oral health-related quality of life. These findings indicate that periodontal health is not limited to oral tissues but is also closely associated with an individual’s overall well-being.

## 1. Introduction

Periodontal diseases are chronic inflammatory conditions with a high prevalence that affect the gingiva and supporting tissues and represent a significant public health problem that can influence not only oral health but also overall health [1]. In the pathogenesis of these diseases, the host immune-inflammatory response to dental plaque plays a decisive role, and the balanced regulation of this response is critical for the maintenance of periodontal health [2]. In recent years, accumulating evidence has suggested that periodontitis is associated not only with oral tissues but also with systemic diseases. In particular, a potential association between periodontitis and cardiovascular diseases has been proposed, mediated through mechanisms such as chronic inflammation and endothelial dysfunction. However, the underlying mechanisms of this association have not yet been fully elucidated [3,4].

In recent years, the impact of systemic factors on periodontal diseases has attracted increasing attention. In particular, sleep disorders and poor sleep quality have been shown to adversely affect immune system functions, increase proinflammatory cytokine levels, and render individuals more susceptible to infections. Sleep disturbances may contribute to periodontal disease progression through several biological pathways, including increased systemic inflammation, elevated levels of proinflammatory cytokines such as interleukin-6 (IL-6) and tumor necrosis factor-alpha (TNF-α), and alterations in pain perception. Additionally, periodontal inflammation-related discomfort and pain may prolong sleep latency and impair overall sleep quality. These biological mechanisms are thought to play a role in the development and progression of periodontal diseases [5,6,7]. A systematic review conducted by Muniz et al. [8] revealed that this relationship is not clearly established and that the existing findings are heterogeneous. This situation indicates that the relationship between sleep quality and periodontal disease should be evaluated through more comprehensive and multidimensional approaches.

The Pittsburgh Sleep Quality Index (PSQI), which is widely used to assess sleep quality, is a valid and reliable self-report instrument that evaluates individuals’ sleep status over the previous month. The Turkish version of the PSQI has been widely used in studies assessing sleep quality in the Turkish population [9]. The PSQI consists of seven components, with a global score ranging from 0 to 21. A total score of 5 or higher indicates poor sleep quality, and higher scores reflect poorer sleep quality [10]. However, the diagnosis of sleep disorders is based on the International Classification of Sleep Disorders, Third Edition, Text Revision (ICSD-3-TR) [11]. While insomnia is primarily diagnosed according to clinical criteria, polysomnography (PSG) is considered the gold standard for the diagnosis of obstructive sleep apnea (OSA) [12]. In recent years, increasing attention has also been directed toward comorbid insomnia and obstructive sleep apnea (COMISA), emphasizing the importance of comprehensive evaluation and management of coexisting sleep disorders [13].

On the other hand, scientific evidence indicating that periodontal diseases negatively affect oral health-related quality of life has been steadily increasing [14]. One of the most commonly used instruments for assessing this outcome, the Oral Health Impact Profile-14 (OHIP-14), has been shown to be valid and reliable across different populations [15]. The Turkish version of the OHIP-14 is widely used to evaluate oral health-related quality of life. The total OHIP-14 score ranges from 0 to 56, with higher scores indicating a greater negative impact on oral health-related quality of life [16].

This study aimed to evaluate sleep quality and oral health-related quality of life in periodontally healthy individuals and those with gingivitis and periodontitis and to investigate the relationship between these parameters.

## 2. Materials and Methods

### 2.1. Ethics Committee Approval

This study was approved by the Clinical Research Ethics Committee of Dicle University with the decision dated 26 March 2025 and numbered 2025/24.

### 2.2. Scope of the Study

The study was conducted between April 2025 and October 2025 on volunteer individuals who presented to the Department of Periodontology at Dicle University Faculty of Dentistry for periodontal examination and treatment. This was a prospective cross-sectional study, and all clinical examinations and questionnaire data were collected prospectively during the study period. A total of 195 participants were included in the study. All individuals were provided with detailed information about the study and gave written informed consent before participation.

### 2.3. Inclusion and Exclusion Criteria

The inclusion criteria were defined as being over 18 years of age, having at least 16 teeth, having no physical or mental disability, not having received periodontal treatment within the last 6 months, and having a panoramic radiograph taken within the last 6 months. The exclusion criteria were defined as pregnancy or lactation, a history of sleep apnea, presence of systemic disease, antibiotic use within the last 3 months, and the presence of significant psychosocial factors that could affect sleep quality (such as unemployment, depression, bankruptcy, or bereavement).

### 2.4. Formation of Study Groups

The study was conducted in three groups: healthy (*n* = 65), gingivitis (*n* = 65), and periodontitis (*n* = 65). According to the power analysis, a minimum of 57 participants per group (a total of 171) was determined to be sufficient at a 95% power level; however, to compensate for potential exclusions and to ensure adequate statistical power, a total of 195 patients were included in the study.

The periodontal status of the participants was determined according to the 2017 classification of periodontal diseases and conditions.

The healthy group was evaluated in two subgroups: periodontal health in an intact periodontium and periodontal health in a reduced periodontium. In both conditions, individuals with bleeding on probing ≤ 10%, probing depth ≤ 3 mm, and no signs of active inflammation were included.

The gingivitis group was divided into two subgroups: gingival inflammation in an intact periodontium and gingival inflammation in a reduced periodontium. Gingival inflammation in a reduced periodontium included individuals without clinical attachment loss or those with a history of attachment loss but no current signs of active periodontitis, presenting with probing depth ≤ 3 mm and bleeding on probing ≥10%.

The periodontitis group consisted of individuals with interdental clinical attachment loss ≥ 1 mm at ≥2 non-adjacent teeth, or buccal/oral clinical attachment loss ≥ 3 mm with concomitant pocket formation at a single tooth. The cases were further classified according to their stage and grade.

### 2.5. Development of the Questionnaire Form

Data were collected through face-to-face questionnaires as well as clinical and radiographic evaluations. The assessment form consisted of four sections: (1) Sociodemographic data: participants’ age, gender, educational level, medication use, smoking habits, and socioeconomic status were recorded; (2) Quality of life: oral health-related quality of life was assessed using the Oral Health Impact Profile-14 (OHIP-14); (3) Sleep quality: sleep quality was evaluated using the Pittsburgh Sleep Quality Index (PSQI); (4) Periodontal evaluation: performed by a single examiner (V.Y) using a calibrated periodontal probe, and plaque index (PI), gingival index (GI), bleeding on probing (BOP), probing pocket depth (PPD), and clinical attachment loss (CAL) were measured and recorded.

Alveolar bone loss was assessed on existing panoramic radiographs by measuring the distance between the cementoenamel junction and the alveolar crest, and these data were used for staging and grading of the disease.

### 2.6. Statistical Analysis

The normality of continuous variables was assessed using the Shapiro–Wilk test. According to the results of the normality test, variables showing normal distribution were expressed as mean ± standard deviation, whereas variables not conforming to normal distribution were presented as median (minimum: maximum); categorical variables were expressed as *n* (%).

Based on the normality results, for comparisons among more than two groups, one-way ANOVA was used when the data were normally distributed, while the Kruskal–Wallis test was applied when normal distribution was not observed. In cases of overall significance following ANOVA, post hoc subgroup analyses were performed using the Bonferroni test; similarly, following a significant Kruskal–Wallis test, subgroup analyses were conducted using the Dunn–Bonferroni test.

The relationships between scales were evaluated using Spearman’s rank correlation analysis due to the non-normal distribution of the data.

To assess the independent association between periodontal status and PSQI and OHIP-14 total scores, two separate multiple linear regression analyses were performed, adjusting for age, sex, education level, and smoking status as potential confounders.

Statistical analyses were performed using SPSS software (IBM Corp., Released 2017. IBM SPSS Statistics for Windows, Version 25.0, IBM Corp., Armonk, NY, USA), and a *p*-value < 0.05 was considered statistically significant.

## 3. Results

When the age distribution of the participants was examined, it was determined that the highest proportion (42.6%) belonged to the 36–59 age group. In terms of gender distribution, 57.9% of the participants were found to be female. According to the evaluation of educational level, the highest proportion (42.1%) was observed in individuals with undergraduate/graduate education. When marital status was assessed, 48.7% of the participants were married and 51.3% were single. Regarding smoking status, 59.5% of the individuals were non-smokers, while 20.0% smoked 1–10 cigarettes per day and 20.5% smoked ≥10 cigarettes per day (Table 1).

When the distribution according to groups was examined, it was observed that 33.3% of the participants were in the healthy group (*n* = 65), 33.3% in the gingivitis group (*n* = 65), and 33.3% in the periodontitis group (*n* = 65). When individuals with periodontitis were evaluated in terms of staging, 16.9% of the cases were Stage 1 (*n* = 11), 35.4% were Stage 2 (*n* = 23), 30.8% were Stage 3 (*n* = 20), and 16.9% were Stage 4 (*n* = 11). In terms of grading, 10.8% of the individuals were Grade A (*n* = 7), 40.0% were Grade B (*n* = 26), and 49.2% were Grade C (*n* = 32) (Table 2).

It was determined that the PI, BOP, GI, and PD values of the healthy group were significantly lower compared to both the gingivitis and periodontitis groups (*p* < 0.001). Additionally, the clinical parameters of the gingivitis group were found to be lower than those of the periodontitis group (*p* < 0.001).

In terms of clinical attachment loss (CAL), the values in the periodontitis group were found to be significantly higher compared to the healthy and gingivitis groups (*p* < 0.001), whereas no significant difference was observed between the healthy and gingivitis groups (*p* > 0.05) (Table 3).

It was determined that the OHIP-14 total score, as well as the subscale scores for functional limitation, physical pain, psychological discomfort, physical disability, psychological disability, social disability, and handicap in the healthy group, were significantly lower compared to both the gingivitis and periodontitis groups (*p* < 0.001). Additionally, the gingivitis group was found to have lower values than the periodontitis group in terms of these parameters (*p* < 0.001) (Table 4).

The PSQI total score of the healthy group was found to be significantly lower compared to both the gingivitis and periodontitis groups (*p* < 0.001 for both comparisons), and the gingivitis group was also found to have a lower score than the periodontitis group (*p* = 0.034).

Lower values were also observed in the healthy group compared to the other groups for the subcomponents of subjective sleep quality and sleep latency. Additionally, the gingivitis group was found to have lower values than the periodontitis group in terms of subjective sleep quality (*p* = 0.017); however, no significant difference was observed between the gingivitis and periodontitis groups for sleep latency (*p* > 0.05).

No statistically significant differences were observed among the groups in terms of the other PSQI subcomponents (sleep duration, habitual sleep efficiency, sleep disturbances, use of sleep medication, and daytime dysfunction) (*p* > 0.05).

It was determined that the OHIP-14 values in the Stage 1 group were significantly lower compared to the Stage 3 and Stage 4 groups (*p* = 0.002 for both comparisons), whereas no significant differences were observed in the other pairwise comparisons (*p* > 0.05).

In terms of PSQI score, no statistically significant difference was observed among the stage groups (*p* = 0.110) (Table 5).

It was determined that the OHIP-14 values in the Grade C group were significantly higher compared to the Grade A and Grade B groups (*p* = 0.026 and *p* = 0.009, respectively), whereas no significant difference was observed between the Grade A and Grade B groups (*p* > 0.05).

In terms of PSQI score, no statistically significant difference was observed among the grade groups (*p* = 0.060) (Table 6).

A statistically significant, positive, and strong correlation was observed between the total PSQI score and the total OHIP-14 score (r = 0.63; *p* < 0.001). It was also determined that the total PSQI score showed moderate and significant correlations with the subdomains of OHIP-14 (r = 0.32–0.53; *p* < 0.001). Among the PSQI components, subjective sleep quality, sleep latency, and sleep disturbances were found to be significantly associated with OHIP-14, whereas no significant relationship was identified with sleep medication use (*p* > 0.05). Overall, deterioration in sleep quality appears to be associated with oral health–related quality of life (Table 7).

Multiple linear regression (Enter method); adjusted for periodontal status, age, sex, education level, and smoking status. * *p* < 0.05.

Multiple linear regression analysis was performed to evaluate the independent effect of periodontal status on PSQI total score, adjusting for age, sex, education level, and smoking status. The model was statistically significant (F(9,185) = 18.50, *p* < 0.001) and explained 44.8% of the variance in PSQI score (Adjusted R^2^ = 0.448). With the healthy group as reference, membership in the gingivitis (B = 3.63, 95% CI: 2.80–4.47, *p* < 0.001) and periodontitis (B = 5.03, 95% CI: 4.07–5.98, *p* < 0.001) groups was significantly associated with an increase in PSQI score. This finding indicates that periodontal disease severity is associated with sleep quality independently of the sociodemographic variables examined. Female sex showed a weak but significant association with PSQI score (B = 0.77, *p* = 0.030). Age, education level, and smoking status were not statistically significant in the model (all *p* > 0.05) (Table 8).

Multiple linear regression (Enter method); adjusted for periodontal status, age, sex, education level, and smoking status. * *p* < 0.05.

In the second model, multiple linear regression analysis was performed to evaluate the independent effect of periodontal status on OHIP-14 total score, adjusting for age, sex, education level, and smoking status. The model was significant (F(9,185) = 33.01, *p* < 0.001) and explained 59.8% of the variance (Adjusted R^2^ = 0.598). Periodontal status emerged as the strongest and most consistent predictor of OHIP-14 score: compared with the healthy group, the gingivitis group scored on average 6.83 points higher (95% CI: 4.85–8.81, *p* < 0.001), and the periodontitis group scored 15.80 points higher (95% CI: 13.54–18.06, *p* < 0.001). This finding demonstrates that the deterioration in oral health-related quality of life increases markedly with increasing periodontal disease severity, and that this association is independent of age, sex, education, and smoking status. In addition, being in the 36–59 age group (B = 2.01, *p* = 0.036) and smoking more than 10 cigarettes per day (B = 2.46, *p* = 0.031) were also significantly associated with OHIP-14 score.

## 4. Discussion

The primary finding of the present study was the progressive deterioration in oral health–related quality of life and sleep quality from healthy individuals to those with gingivitis and periodontitis, suggesting that the presence of periodontal disease is associated with unfavorable patient-reported outcomes. These findings indicate that the impact of periodontal disease may extend beyond local tissue destruction and may also be associated with functional, psychological, and social aspects of well-being. Furthermore, multiple linear regression analyses demonstrated that periodontal status remained significantly associated with both OHIP-14 and PSQI scores after adjustment for age, sex, education level, and smoking status. These findings further suggest that the observed associations between periodontal status and these patient-reported outcomes may be independent of these potential confounding variables.

The differences observed across all OHIP-14 domains among the periodontal groups suggest that the impairment in oral health–related quality of life may follow a multidimensional pattern. In particular, the higher scores in the functional limitation and physical pain domains indicate that periodontal disease may adversely affect daily activities and oral function. These findings are consistent with those reported in previous studies [17,18,19]. Taken together, the present results support the concept that periodontal inflammation should be regarded not only as a biological process but also as a clinical condition with important implications for patient-centered outcomes.

Higher scores in the psychological discomfort and psychological disability domains further suggest that periodontal disease may adversely influence individuals’ emotional well-being and social perception. However, the variability observed among participants indicates that the impact of periodontal disease on quality of life may not be determined solely by disease severity but may also be influenced by factors such as individual perceptions, cultural background, and coping strategies. These findings are consistent with previous studies emphasizing the multifactorial nature of the relationship between periodontal disease and oral health–related quality of life [20,21,22].

Consistent with the existing literature, the findings related to the physical disability and social disability domains suggest that progression of periodontal disease may be associated with reduced functional capacity and impaired social interactions [23]. Likewise, the increase observed in the handicap domain may reflect the potential impact of periodontal disease on overall life satisfaction. In this context, the relatively lower impact reported among individuals receiving supportive periodontal therapy further highlights the importance of regular periodontal maintenance, as previously demonstrated [24,25,26]. Furthermore, the progressive increase in total OHIP-14 scores with increasing disease severity and grade suggests that deterioration in oral health–related quality of life may be cumulative and progressive in nature [27,28,29]. The higher OHIP-14 scores observed in the stage- and grade-based analyses further indicate that patient-centered outcomes may become more adversely affected as periodontal disease progresses.

Nevertheless, previous studies have reported inconsistent findings regarding the association between periodontal disease and overall quality of life. A cross-sectional study conducted among individuals with controlled chronic systemic diseases found no significant association between periodontal status and overall quality of life [30]. These discrepancies may be attributable to differences in the quality-of-life instruments used, characteristics of the study populations, and methodological approaches. In contrast, the present study specifically evaluated oral health–related quality of life using the OHIP-14 questionnaire, providing a more focused assessment of the impact of periodontal disease on patients’ daily lives and oral health experiences.

Regarding sleep quality, significant associations were observed particularly for the subjective sleep quality and sleep latency components of the PSQI. Consistent with previous studies, the progressive deterioration in these parameters from healthy individuals to those with gingivitis and periodontitis suggests that the relationship between periodontal disease and sleep quality may be associated with both the presence and clinical status of periodontal disease [31,32,33]. This association may be explained by periodontal inflammation–related pain, discomfort, and psychological stress, all of which have the potential to disrupt normal sleep patterns.

In contrast, the absence of significant differences in the remaining PSQI components suggests that periodontal disease may not affect all dimensions of sleep quality to the same extent. This finding is in agreement with previous reports indicating that certain sleep parameters may be more strongly associated with periodontal status than others [32,34]. In addition, individual variability and lifestyle-related factors may further contribute to the heterogeneous nature of this relationship.

The progressive increase in total PSQI scores from healthy individuals to those with gingivitis and periodontitis further supports a potential association between poor sleep quality and periodontal disease [35,36]. However, the lack of a significant association between total PSQI scores and periodontitis stage or grade suggests that this relationship may not follow a linear pattern according to disease severity or progression. The inconsistent findings reported in the literature may be explained by differences in study populations, methodological approaches, and individual variability [32,37].

One of the most notable findings of the present study was the strong positive correlation observed between the total PSQI and OHIP-14 scores. Furthermore, the moderate and significant correlations between the total PSQI score and most OHIP-14 domains suggest that deterioration in sleep quality may occur in parallel with impairment in oral health–related quality of life. Significant associations between subjective sleep quality, sleep latency, sleep disturbances, and OHIP-14 scores further support the role of subjective sleep experiences in shaping oral health–related quality of life. In contrast, the absence of a significant association between the use of sleep medication and OHIP-14 scores suggests that not all dimensions of sleep quality are equally related to oral health–related quality of life.

Overall, the relationship between periodontal disease and sleep quality may be influenced by the complex interplay of biological and psychosocial mechanisms. Increased systemic inflammation may contribute to disturbances in sleep patterns, whereas poor sleep quality may, in turn, exacerbate periodontal tissue destruction, suggesting a potentially bidirectional relationship. These findings support the concept that periodontal disease should be considered not only as a condition affecting the oral tissues but also as a complex disorder with systemic and behavioral dimensions.

This study has several limitations that should be considered. First, the cross-sectional design precludes any causal inferences regarding the observed associations. Second, the use of self-reported instruments (PSQI and OHIP-14) may have introduced response bias related to participants’ subjective perceptions. In addition, sleep quality was assessed exclusively using a subjective questionnaire without objective sleep measurements, which should be taken into account when interpreting the findings. Furthermore, although all periodontal clinical measurements were performed by an experienced periodontist, intra-examiner reliability was not assessed, and this should be considered when interpreting the clinical measurements. The single-center design and the relatively specific study population may also limit the generalizability of the findings. Nevertheless, the present study contributes to a better understanding of the association between periodontal disease, sleep quality, and oral health-related quality of life in a systemically healthy population. Therefore, future studies with larger sample sizes, multicenter settings, longitudinal designs, and assessment of intra-examiner reliability are warranted to validate the present findings, evaluate their generalizability to different populations, and provide a more comprehensive and methodologically robust understanding of these associations.

## 5. Conclusions

In conclusion, the findings of the present study suggest that periodontal disease may extend beyond local tissue destruction and may also be associated with oral health-related quality of life and sleep quality. The progressive increase in OHIP-14 and PSQI scores from healthy individuals to those with gingivitis and periodontitis indicates that these associations may be clinically relevant. Furthermore, the significant correlation observed between sleep quality and oral health-related quality of life supports the potential link between periodontal disease and patient-centered outcomes. Therefore, incorporating patient-centered measures, such as oral health-related quality of life and sleep quality, alongside conventional clinical parameters may contribute to a more comprehensive assessment and management of periodontal disease.

## Figures and Tables

**Table 1:** Distribution of Participants’ Demographic Characteristics and Their Distribution According to Periodontal Health Groups.

Variable	Category	*n* (%)
Age	18–35 years	80 (41)
	36–59 years	83 (42.6)
	≥60 years	32 (16.4)
Gender	Female	113 (57.9)
	Male	82 (42.1)
Education level	Illiterate–Primary school	41 (21)
	Secondary education–High school	72 (36.9)
	Undergraduate–Postgraduate	82 (42.1)
Marital status	Married	95 (48.7)
	Single	100 (51.3)
Smoking status	Non-smoker	116 (59.5)
	1–10 cigarettes/day	39 (20)
	≥10 cigarettes/day	40 (20.5)
Group	Healthy	65 (33.3)
	Gingivitis	65 (33.3)
	Periodontitis	65 (33.3)
Stage	Stage 1	11 (16.9)
	Stage 2	23 (35.4)
	Stage 3	20 (30.8)
	Stage 4	11 (16.9)
Grade	Grade A	7 (10.8)
	Grade B	26 (40)
	Grade C	32 (49.2)

The data are expressed as *n* (%).

**Table 2:** Comparison of Participants’ Indices According to Periodontal Health Groups.

Parameter	Healthy (*n* = 65)	Gingivitis (*n* = 65)	Periodontitis (*n* = 65)	*p*-Value
PI	0.58 (0.26–0.95); 0.59 ± 0.17	1.55 (1.04–2.32); 1.57 ± 0.28	1.95 (1.27–2.80); 2.03 ± 0.37	<0.001 ^a^
BOP (%)	5.36 (3.25–8.04); 5.35 ± 1.18	31.32 (16.14–49.31); 31.33 ± 9.50	51.47 (30.13–82.46); 51.48 ± 11.73	<0.001 ^a^
GI	0.6 (0.26–0.97); 0.61 ± 0.17	1.6 (1.02–2.37); 1.61 ± 0.27	1.97 (1.45–2.78); 2.06 ± 0.34	<0.001 ^a^
PD (mm)	1.86 (1.20–2.15); 1.85 ± 0.16	2.08 (1.45–2.67); 2.07 ± 0.27	3.32 (2.3–5.21); 3.39 ± 0.56	<0.001 ^a^
CAL (mm)	0 (0–3.47); 1.01 ± 1.27	0 (0–3.56); 0.73 ± 1.3	2.6 (1.78–4.03); 2.71 ± 0.55	<0.001 ^a^

Data are presented as median (minimum–maximum) and mean ± standard deviation. ^a^: Kruskal–Wallis test.

**Table 3:** Comparison of OHIP-14 and Its Subscales According to Periodontal Health Groups.

Parameter	Healthy (*n* = 65)	Gingivitis (*n* = 65)	Periodontitis (*n* = 65)	*p*-Value
OHIP-14	6 (0–18); 6.14 ± 4.16	13 (3–25); 12.82 ± 5.24	22 (5–37); 22.29 ± 6.92	<0.001 ^a^
Functional Limitation	0 (0–4); 0.49 ± 0.89	1 (0–4); 1.22 ± 1.09	2 (0–6); 2.28 ± 1.62	<0.001 ^a^
Physical Pain	1 (0–4); 1.00 ± 1.13	2 (0–6); 2.49 ± 1.47	4 (1–8); 3.80 ± 1.73	<0.001 ^a^
Psychological Discomfort	2 (0–6); 2.06 ± 1.89	2 (0–8); 2.75 ± 1.74	4 (0–8); 3.85 ± 1.69	<0.001 ^a^
Physical Disability	0 (0–5); 0.75 ± 1.10	2 (0–6); 1.83 ± 1.49	3 (0–6); 3.20 ± 1.59	<0.001 ^a^
Psychological Disability	0 (0–5); 0.65 ± 0.95	2 (0–4); 1.57 ± 1.21	3 (0–6); 3.00 ± 1.40	<0.001 ^a^
Social Disability	0 (0–4); 0.74 ± 1.09	1 (0–7); 1.68 ± 1.48	3 (0–6); 3.18 ± 1.66	<0.001 ^a^
Handicap	0 (0–4); 0.45 ± 0.83	1 (0–4); 1.28 ± 1.11	3 (0–8); 2.98 ± 1.79	<0.001 ^a^

Data are presented as median (minimum–maximum) and mean ± standard deviation. ^a^: Kruskal–Wallis test.

**Table 4:** Comparison of PSQI and Its Subscales According to Periodontal Health Groups.

Parameter	Healthy (*n* = 65)	Gingivitis (*n* = 65)	Periodontitis (*n* = 65)	*p*-Value
PSQI	4 (2–14); 4.77 ± 2.11	8 (4–17); 8.37 ± 2.29	10 (3–14); 9.72 ± 2.52	<0.001 ^a^
Subjective Sleep Quality	1 (0–3); 0.85 ± 0.73	1 (0–3); 1.38 ± 0.70	2 (0–3); 1.75 ± 0.71	<0.001 ^a^
Sleep Latency	1 (0–3); 0.69 ± 0.66	1 (0–3); 1.40 ± 0.73	2 (0–3); 1.63 ± 0.76	<0.001 ^a^
Sleep Duration	1 (0–2); 0.82 ± 0.56	1 (0–3); 1.31 ± 0.75	1 (0–3); 1.46 ± 0.83	>0.99 ^a^
Habitual Sleep Efficiency	1 (0–2); 0.60 ± 0.55	1 (0–3); 1.29 ± 0.82	1 (0–3); 1.45 ± 0.73	>0.99 ^a^
Sleep Disturbances	1 (0–2); 0.83 ± 0.45	2 (0–3); 1.55 ± 0.77	2 (1–3); 1.71 ± 0.58	>0.99 ^a^
Use of Sleep Medication	0 (0–1); 0.34 ± 0.48	0 (0–3); 0.35 ± 0.59	0 (0–3); 0.42 ± 0.73	>0.99 ^a^
Daytime Dysfunction	1 (0–2); 0.65 ± 0.59	1 (0–3); 1.08 ± 0.67	1 (0–3); 1.31 ± 0.61	>0.99 ^a^

Data are presented as median (minimum–maximum) and mean ± standard deviation. ^a^: Kruskal–Wallis test.

**Table 5:** Comparison of OHIP-14 and PSQI Scores According to Periodontitis Stage and Grade.

Parameter	Stage 1 (*n* = 11)	Stage 2 (*n* = 23)	Stage 3 (*n* = 20)	Stage 4 (*n* = 11)	*p*-Value
OHIP-14	17 (5–24); 16.18 ± 5.06	19 (13–37); 20.57 ± 6.13	24 (16–37); 25.05 ± 5.21	26 (13–37); 27.00 ± 7.69	<0.001 ^a^
PSQI	8.64 ± 1.96	9.22 ± 2.56	10.35 ± 1.87	10.73 ± 3.44	0.110 ^b^
Parameter	Grade A (*n* = 7)	Grade B (*n* = 26)	Grade C (*n* = 32)		*p*-value
OHIP-14	17 (5–29); 17.29 ± 7.52	18 (12–37); 20.15 ± 6.06	24 (13–37); 25.13 ± 6.36		0.002 ^a^
PSQI	9.86 ± 2.41	8.85 ± 1.95	10.41 ± 2.78		0.060 ^a^

Data are presented as median (minimum–maximum) and mean ± standard deviation. ^a^: Kruskal–Wallis test, ^b^: ANOVA test.

**Table 6:** Correlation Between the PSQI and OHIP-14 Scales.

		OHIP-14	Functional Limitation	Physical Pain	Psychological Discomfort	Physical Disability	Psychological Disability	Social Disability	Handicap
PSQI	r_s_	0.63	0.36	0.52	0.32	0.47	0.49	0.53	0.49
	p	<0.001 *	<0.001 *	<0.001 *	<0.001 *	<0.001 *	<0.001 *	<0.001 *	<0.001 *
Subjective Sleep Quality	r_s_	0.49	0.35	0.36	0.31	0.39	0.34	0.37	0.40
	p	<0.001 *	<0.001 *	<0.001 *	<0.001 *	<0.001 *	<0.001 *	<0.001 *	<0.001 *
Sleep Latency	r_s_	0.51	0.27	0.46	0.31	0.42	0.33	0.37	0.41
	p	<0.001 *	<0.001 *	<0.001 *	<0.001 *	<0.001 *	<0.001 *	<0.001 *	<0.001 *
Sleep Duration	r_s_	0.33	0.21	0.26	0.12	0.23	0.30	0.34	0.22
	p	<0.001 *	0.003 *	<0.001 *	0.094	<0.001 *	<0.001 *	<0.001 *	0.002 *
Habitual Sleep Efficiency	r_s_	0.38	0.24	0.32	0.19	0.23	0.30	0.35	0.30
	p	<0.001 *	0.001 *	<0.001 *	0.008 *	0.001 *	<0.001 *	<0.001 *	<0.001 *
Sleep Disturbances	r_s_	0.51	0.29	0.39	0.19	0.38	0.40	0.43	0.41
	p	<0.001 *	<0.001 *	<0.001 *	<0.001 *	<0.001 *	<0.001 *	<0.001 *	<0.001 *
Use of Sleep Medication	r_s_	−0.06	−0.09	−0.04	−0.07	−0.09	0	0	0
	p	0.380	0.190	0.614	0.326	0.216	0.993	0.973	0.646
Daytime Dysfunction	r_s_	0.36	0.17	0.26	0.17	0.31	0.32	0.32	0.29
	p	<0.001 *	0.021 *	<0.001 *	0.018 *	<0.001 *	<0.001 *	<0.001 *	<0.001 *

r_s_: Spearman’s correlation coefficient. * indicates statistical significance at *p* < 0.05.

**Table 7:** Multiple Linear Regression Analysis—PSQI Total Score.

Variable	B	SE	β	95% CI	*p*-Value
Periodontal status, ref: Healthy					
Gingivitis	3.63	0.42	0.55	2.79 to 4.47	<0.001 *
Periodontitis	5.03	0.48	0.76	4.07 to 5.98	<0.001 *
Age, (ref: 18–35)					
36–59	−0.1	0.4	−0.02	−0.89 to 0.69	0.803
60>	−0.36	0.56	−0.04	−1.47 to 0.76	0.528
Sex: Female ref: Male	0.77	0.35	0.12	0.07 to 1.47	0.030 *
Education, ref: Illiterate-Primary school					
Secondary-High school	−0.12	0.49	−0.02	−1.10 to 0.86	0.809
Bachelor-Postgraduate	−0.06	0.54	−0.01	−1.13 to 1.00	0.903
Smoking, ref: No					
1–10 cigarettes/day	−0.31	0.48	−0.04	−1.26 to 0.63	0.514
>10 cigarettes/day	0.32	0.48	0.04	−0.62 to 1.26	0.502

R^2^ = 0.474, Adjusted R^2^ = 0.448, Model *p*-value ≤ 0.001, B: unstandardized regression coefficient; SE: standard error; β: standardized regression coefficient; CI: confidence interval; ref: reference category. * indicates statistical significance at *p* < 0.05.

**Table 8:** Multiple Linear Regression Analysis—OHIP-14 Total Score.

Variable	B	SE	β	95% CI	*p*-Value
Periodontal status, ref: Healthy					
Gingivitis	6.83	1	0.37	4.85 to 8.81	<0.001 *
Periodontitis	15.8	1.15	0.86	13.54 to 18.06	<0.001 *
Age, ref: 18–35					
36–59	2.01	0.95	0.11	0.14 to 3.88	0.036 *
60>	2.11	1.34	0.09	−0.54 to 4.75	0.118
Sex: Female (ref: Male)	0.78	0.84	0.04	−0.88 to 2.44	0.355
Education, ref: Illiterate-Primary school					
Secondary-High school	−0.07	1.17	0	−2.38 to 2.25	0.955
Bachelor-Postgraduate	2.06	1.28	0.12	−0.47 to 4.59	0.109
Smoking, ref: No					
1–10 cigarettes/day	0.15	1.14	0.01	−2.09 to 2.39	0.893
>10 cigarettes/day	2.46	1.13	0.11	0.23 to 4.70	0.031 *

R^2^ = 0.616, Adjusted R^2^ = 0.598, Model *p*-value ≤ 0.001, B: unstandardized regression coefficient; SE: standard error; β: standardized regression coefficient; CI: confidence interval; ref: reference category. * indicates statistical significance at *p* < 0.05.

## Data Availability

The data presented in this study are available from the corresponding author upon reasonable request. The data are not publicly available due to privacy and ethical restrictions.

## Abbreviations

The following abbreviations are used in this manuscript:

BOP	Bleeding on Probing
PSQI	Pittsburgh Sleep Quality Index
PI	Plaque Index
CAL	Clinical Attachment Loss
OHIP-14	Oral Health Impact Profile-14
PD	Probing Depth
